# Framing the failure of medical implants: Media representations of the ASR hip replacements in the UK

**DOI:** 10.1111/hex.12877

**Published:** 2019-03-19

**Authors:** Gregory Maniatopoulos, Clare Hopkins, Thomas J Joyce, Katie Brittain

**Affiliations:** ^1^ Institute of Health and Society Newcastle University Newcastle upon Tyne UK; ^2^ School of Engineering Newcastle University Newcastle upon Tyne UK; ^3^ Department of Nursing, Midwifery and Health Northumbria University Newcastle upon Tyne UK

**Keywords:** media framing, media representations, medical device, medical implant failure, metal‐on‐metal hips, United Kingdom

## Abstract

**Background:**

During the twentieth century, hip replacement became one of the most popular and successful operations. In the 1990s, a new type of hip replacement namely the metal‐on‐metal hip resurfacing was developed. This paper draws on one of the available implants, namely the DePuy Orthopaedics’ Articular Surface Replacement (ASR) hip system which was withdrawn from the market because of higher than expected rates of failure. It examines media representations on the failure of the ASR metal‐on‐metal hip replacement device and its subsequent withdrawal from the market.

**Methods:**

Drawing on content analysis this paper explores how systemic failure of the medical implant was framed and performed by press media in the UK.

**Results:**

Two narratives were particularly important in framing press media coverage of the ASR case: the role of patients as passive recipients of care and a distinction between health and disability identities as related to how individuals’ narratives about the past shaped their sense of present and future. In all cases, the voice of the orthopaedic surgeons responsible for the selection and implantation of the ASR devices remains silent.

**Conclusions:**

Press media coverage of medically induced harm in the UK is significantly less common than coverage of any other patient safety issues and public health debates. This study aims to contribute to the evidence base on how public discourse on medically induced harm becomes framed through the reported experiences of individuals in press media and also how this process influences the legitimacy of various solutions to medical errors or unanticipated outcomes.

## 1 INTRODUCTION

This paper examines media representations that shape instances of iatrogenic harm that occur as a result of a medical intervention, specifically hip replacements. lllich^1^ coined the term “iatrogenesis” to describe “the undesirable side‐effects of approved, mistaken, callous or contra‐indicated technical contacts with the medical system” (p. 41). An investigation in the UK^2^ estimated that medically induced harm to patients occurred in more than 850 000 cases a year (10% of hospital admissions). The subject of iatrogenic harm occurring as a result of hip replacement is important because of the increasing number of people who require treatment for the effects of diseased or damaged hips. Recent reports illustrate that between 2003 and 2017, 890 681 primary hip replacements were carried out in England, Wales and Northern Ireland.^3^ In this context, hip replacement came to be viewed as one of the most popular operations of the twentieth century.^4^ However, it frequently becomes an impatiently awaited operation for those experiencing increasing pain and life‐disrupting loss of function.^5^

This paper draws on one of the available implants namely the DePuy Orthopaedics’ Articular Surface Replacement (ASR) hip system which was withdrawn from the market because of higher than expected rates of failure. Drawing on content analysis, it explores how systemic failure of the medical implant was framed and performed by press media in the United Kingdom (UK). In so doing, we aim to explore the reported experiences of damaged hip recipients and the positioning of other key players (ie news actors appearing in the news) who feature in the story of the ASR hip system. Understanding these accounts may illuminate how the experiences of damaged hip recipients were played out in the media and how these representations might influence public perception of medically induced harm. Our aim is to contribute to the evidence base on how public discourse on medically induced harm becomes framed through the reported experiences of individuals in press media and also how this process influences the legitimacy of various solutions to medical errors or unanticipated outcomes.

This paper is organized as follows. The next section summarizes the relationship between media framing and health. Subsequent sections provide a brief overview of the history of hip replacement together with a summary of the DePuy ASR metal‐on‐metal hip case study. The next section describes the methods of the study. The main findings are then presented and discussed. The paper concludes with a discussion about the role of the UK press media in framing medically induced harm through the reported experiences of damaged hip recipients.

## 2 MEDIA FRAMING AND HEALTH

The news media and in particular print media is a strong cultural influence in the UK. They serve as powerful modes of communication and message delivery across a large, anonymous, diverse audience.^6^ This power plays a key role towards the framing of a perceived social reality through the creation of “regimes of truth” which underpin much of what people understand of events that occur around the world on a daily basis (ie what people know or claim to know).^7^, ^8^ In sociology and journalism studies, the concept of framing has been used to explore how media and audiences become mutually embedded in the social construction of news events.^9^ Goffman explored first the role of framing in communication as cognitive structures that guide public perception and the representation of social reality.^10^ He defined frames as a “schemata of interpretation that enable individuals’ to locate, perceive, identify and label occurrences within their life space and their world at large” (p. 464).^11^ However, as cognitive structures, frames not only allow/enable the representation of a perceived social reality but may also constrain/limit versions of it. Entman^12^ argues that “to frame a communicating text or message is to promote certain facets of a ‘perceived reality’ and make them more salient in such a way that endorses a specific problem definition, causal interpretation, moral evaluation, and/or a treatment recommendation” (p. 51). In other words, the process of framing embeds also the reference to some silent aspects of perceived reality.^13^, ^14^, ^15^ By selecting which aspects/issues of the narrative to highlight or to omit news media have the potential to influence public perception and representations of social reality.

In the context of health care, news media provide a strategy through which health messages are delivered.^16^ They play a critical role in shaping public opinion of, and willingness to accept, new health interventions but also public policy formation for various health‐related issues.^17^, ^18^, ^19^, ^20^, ^21^, ^22^ In so doing, news media are active in setting the frames of reference that public use to construct meanings and representations of health‐related behaviour, health‐care utilization and health‐care practices.^14^, ^16^, ^23^ These include representations of what it is like to be sick, what causes illness, health and cure, how providers deliver and evaluate health‐care services and interventions.^16^ In this context, it has been suggested that news media “may increase or diminish the willingness of individuals to present themselves for care, and raise expectations, and dash hopes, or may provoke alarm” (p. 7).^24^ Apart from the general public, news media could also affect policy makers’ and health‐care professionals’ perception and awareness of health‐related issues and influence their practice.^23^ For example, in the context of health policy formation, it has been suggested that media frames have the potential “to affect the nature of regulation, the course of litigation, or the direction of research and development” (p. 54).^25^

In this context, through the framing process, news media have the ability to define a health problem and its causes but also could influence the legitimacy of various solutions.^12^ Recent studies have explored the critical role of media framing on various health‐related issues such as breast cancer, obesity, abortion debates, the outbreak of a new virus and severe infectious disease.^26^, ^27^, ^28^, ^29^, ^30^, ^31^, ^32^ Despite the high prevalence of medically induced harm, research on the media framing of such incidents is more limited.^33^ Considerable benefits may result from exploring news media coverage of medically induced harm to encourage support for evidence‐based changes to relevant medication policies and practices. In this context, exploring the ways in which medically induced harm is framed is critical to how different audiences (policy makers and patients) understand and evaluate issues related to harm created by the practice of medicine. This paper aims to explore the ways in which experiences of damaged hip recipients were framed in the media in order to provide a better understanding of how medically induced harm is defined and the solutions offered to counter such events.

## 3 HIP REPLACEMENT: A BRIEF HISTORY

In order to understand the development and popularity of hip replacements, it is necessary to look at how they developed and became embedded within medical practice. The earliest attempts at designing hip replacements were made in the late 1800s but it took until the 1950s before more successful designs began to emerge. Such early prosthesis design was often by orthopaedic surgeons who collaborated with manufacturers over matters of production but retained control over the use of their design, frequently restricting it to older people, less likely to survive longer than the expected life of the prosthesis.^34^ Hip replacement operations became more successful, popular and mainstream throughout the second half of the twentieth century and with this popularity emerged the demand for an implant suitable for younger people wishing to resume an active lifestyle. This prompted a search for new designs and materials capable of overcoming the wear associated with increased activity.^35^ Metal‐on‐metal hip resurfacing was developed during the 1990s in response to this problem and early follow‐up studies were encouraging with many participants subsequently able to resume an active lifestyle.^36^ Among many benefits, the core advantage cited for the use of metal‐on‐metal (cobalt chromium alloy) hip resurfacings with smooth bearing surfaces was that it presented a lowered likelihood of wear.

## 4 THE STORY OF THE ARTICULAR SURFACE REPLACEMENT (ASR)

Several manufacturers marketed metal‐on‐metal hip resurfacing designs and among these were DePuy (a subsidiary of the pharmaceutical company Johnson & Johnson) who developed the Articular Surface Replacement (ASR). The full story of the ASR and the adverse effects that resulted from its implantation, are described fully elsewhere.^37^, ^38^ In summary, where problems occurred, ASR‐recipients began to experience unexplained pain that was subsequently found to be linked to high levels of minute metal particles in their blood caused by wear between the two metal surfaces of the ASR hip. Blood testing subsequently showed that the resurfaced hip might be failing even if the recipient experienced no pain.^39^ High levels of cobalt and chromium in the blood have been associated with inflammatory reactions around the resurfaced joint and may also be associated with neurological and endocrine symptoms.^40^ Table 1 provides a summary of the history of the ASR.

**Table 1:** History of the ASR

2003	ASR resurfacing introduced
2007	High revision rates reported by Australian Orthopaedic Association, National Joint Replacement Registry
2008	Subsequent reporting of high revision rates by Australian Orthopaedic Association, National Joint Replacement Registry
2009	First report of high revision rates in National Joint Registry of England and Wales
	ASR withdrawn in Australia and New Zealand
2010	MHRA issues MDA on all MoM implants noting a small number of patients have adverse reactions
	DePuy release guidance on positioning
	MHRA issue MDA on positioning
	DePuy withdraw the ASR globally

The ASR was withdrawn from use in Australia in late 2009, then withdrawn by the manufacturer in the rest of the world and the UK in August 2010 followed by a recall by the Medicines and Healthcare Products Regulatory Agency (MHRA) in September 2010. It is estimated that 60 000 patients in England and Wales have received metal‐on‐metal hip implants since 2003^41^ with approximately 10 000 of these receiving an ASR. In 2006, 10.8% of all hip replacements were resurfacings but by 2016 this had declined to just 0.7%.^3^ By August 2010, it was recognized that ASR hips had high revision rates. Current revision rates of the ASR are 44% at 10 years. 42 The nice guidance suggests revision rates should be <5% at 10 years.^43^

## 5 METHOD

Drawing upon content analysis,^44^ this study analysed coverage of the DePuy Orthopaedics’ ASR hip system in four UK daily newspapers. The period studied was between August 2010, when the ASR was withdrawn by the manufacturer, and the end of March 2014 when reports related to the ASR had become less prominent. Tabloid newspapers were represented by The Daily Mail/Mail‐online and The Mirror and broadsheets by The Telegraph/Telegraph online and The Independent, giving a wide range of political alignment, editorial approach and readership profile.

A search of the news database LexisLibrary using the terms “DePuy” and “ASR” for the 43 month period yielded 39 texts across a range of tabloid and broadsheet publications. Careful scrutiny of each text revealed that 13 contained “patient stories”: eight of these texts came from three tabloid publications (The Daily Mail, Mailonline and The Mirror) and five from two broadsheet publications (The Telegraph/Telegraph online and The Independent). Twelve ASR‐recipients subsequently requiring revision are presented in these 13 reports (see Table 2). Nine are women and three are men.

**Table 2:** Number of articles and publication

Year	Title	Articles per year	Publication format
2010	The Independent	1	Broadsheet
	Mailonline	1	Tabloid
2011	Mailonline	4	Tabloid
	The Mirror	1	Tabloid
2012	The Sunday Telegraph	1	Broadsheet
	Mailonline	1	Tabloid
	The Independent	1	Broadsheet
	Telegraph online	1	Broadsheet
2013	Telegraph online	1	Broadsheet
	Mailonline	1	Tabloid
Total		13	

To develop a coding frame, all thirteen texts were read by one of the authors (CH) in order to establish the key similarities and differences contained within them as well as identifying repeated/significant language use. These were later discussed with the research team (GM and KB). Files containing the texts were then imported into an Nvivo 10 (QSR International, 2012) software package, and the categories identified through the initial reading used as a coding guide. Due to the diversity of the materials in the texts and because, in each text, a natural division occurred between the personal accounts of hip recipients and the editorial “voice” situating their account within a wider context, the codes were split into two sections. Overall, five key themes emerged. Three of these related to the creation of identities for harmed hip recipients: (a) construction of a passive identity (b) construction of health versus disability identities and (c) construction of victimhood. A further two themes related to the construction and positioning of the other key players in the case of the ASR: (a) the construction of blame and accountability and (b) media‐given voice. For confidentiality reasons, all individuals participating in newspapers reports on the ASR case study have been anonymized. Ethical approval was not required for this study.

## 6 FINDINGS

Five key themes were identified through the analysis, and these are presented in two sections, the first relating to the construction of damaged hip recipients identities and the second relating to the positioning of the other key players in the story surrounding the failure of the ASR.

## 7 CONSTRUCTION OF THE IDENTITIES OF HIP RECIPIENTS

The approach to the construction of ASR‐recipients’ identities was similar across all the texts. Most were constructed as unexceptional and “ordinary” and in only two cases were their previous public identities given prominence: a male government minister, who had previously pursued a military career; and a business woman, previously a gymnast who had become a “poster girl” for the manufacturer before her resurfaced hip joint became problematic.

### 7.1 Construction of a passive “patient” identity

Harmed ASR‐recipients were depicted as largely passive and willing to accept the advice of surgeons treating them. Only one person had carried out any independent research into the ASR prior to its insertion. Others talked of receiving advice from the surgeon and the words attributed to them demonstrate some understanding that the ASR—spoken of by one recipient as “the latest state‐of‐the‐art option”—was being recommended because of their (young) age. This passivity was not problematized in the texts: hip recipients were portrayed as in no position to make demands relating to their treatment or to question the information they received.

This passive patient role within medical encounters is deeply embedded within societal representations of health and illness and is “legitimized in every interaction between patient and health ‘professionals’” (p. 24).^45^ The act of seeking medical help signals an acceptance that the body has become the “territory” of medicine, and is itself an act of passivity. Passive patients, who receive and act unquestioningly on medical advice, may be viewed as “good patients,” defined by Jeffrey^46^ as those who have become unwell through no fault of their own, see their illness as undesirable, allow the doctor to practice their expertize and are willing to cooperate with any help offered. However, passivity may result in poor health care if it also stifles complaint.^47^

### 7.2 Construction of polarized health and disability identities

Damaged hip recipients were further depicted as having polarized health and disability identities, with an emphasis being placed on their previous active lives. They are described as walking, cycling, being “sporty” and generally active. One is quoted as saying:

I was in my late fifties and, apart from some rheumatoid arthritis, I was fit and active at the time my surgeon recommended that I should have hip replacement surgery

In each case, these previous abilities were juxtaposed with the losses encountered as a result of the failure of the ASR. In particular, hip recipients were framed as no longer able to accomplish everyday activities such as dog‐walking or shopping, and in one case no longer being “the hard working, vivacious woman I used to be.” Where the reports remain silent about a hip‐recipient's previous activity levels it is possible to conjecture that these were, in fact, unremarkable. However, this silence effectively contributes towards the construction of a collective active identity for all hip recipients. For only three people is explicit mention made of poor previous/childhood joint health, two having juvenile arthritis and one with joints worn through exercise.

This technique of “exploiting oppositions” (p. 518)^16^ is a common discursive method adopted by news media towards the creation of easily understood polarities and, in this case, identities. Such accounts, constructed selectively, serve not just to frame the representation of active and healthy identities but also to structure a polarity with post‐ASR‐failure bodies. However, they neglect to attend to the factors that necessitated the resurfacing or replacement of their hips that would contradict the constructed active identities.

For those hip recipients without previous public identities, the texts emphasize their “ordinariness” through the use of small personal and family details to which the reader is invited to relate. This emphasis on the “sick” selves of hip recipients limits readers’ understandings of their total experience and perhaps also contributes to the cultural construction of the reader's own identity given that in looking at the lives of others we are “looking always in relation to ourselves” (p. 79).^48^ Jeffrey^46^ suggests that “illness is a morally ambiguous condition” (p. 105) where there is a necessity for the patient to prove that s/he is not deviant. These accounts of hip recipients’ identities as previously healthy and active, leading in most cases, “ordinary” unexceptional lives, constitute them as “genuine” but also further contribute to frame them as victims of iatrogenic harm.

### 7.3 Construction of victimhood

The victimhood of hip recipients is constructed tacitly through the language used to describe the pain they attribute to the failure of the ASR and through descriptions of their emerging recognition of problems with their artificial hip. This awareness unfolded in parallel with increases in the severity of pain and the longevity of the problem: some people knew that something was wrong immediately and for some, the realization of the true impact of the damage became evident only following remedial surgery. This was then followed by the onset of disability and the realization of losses (physical, social and psychological) culminating in a sense of uncertainty and fear about potential future harms.

Almost straight away, I could feel it moving

Great emphasis was placed on the ability of ASR‐recipients to discern problems through attentiveness to the functioning of their bodies and their levels of pain even when no cause for their symptoms could be established. The presence of pain often represented the first indication of a problem and emotive “pain language” such as “constant,” “awful,” “agony,” “crippling” was used as shorthand for the severity of the situation each faced, at the same time emphasizing their vulnerability and passivity. In only one instance is a person shown to be actively resisting pain by measures other than passively taking analgesics.

The helplessness of being in pain without an explanation readily recognizable to others, particularly the medical profession, accentuates vulnerability. The X‐rays of one person's hip showed no abnormality and “surgeons insisted nothing was wrong” until, due to her persistence, the high level of metal ions in her blood was established. Another recounted the experience of presenting at A & E because she was unable to weight‐bear:

[…] but it wasn’t dislocated and no one could find a cause. I spent the next two years in and out of hospital but it wasn’t until I got a second opinion that a product defect was mentioned

Pain is never a purely physical experience but one shaped by emotional, psychological and cultural components.^49^ Despite its universality, pain is hard to describe in a manner comprehensible to others and this may result in feelings of frustration should this inability lead to health professionals’ disbelief.^50^ When accounts of pain are rejected as untrue or inconsequential, a potential exists for this disbelief to be experienced as a “moral event.” Medical disbelief may lead to a sense of narrative disruption for the individual and perhaps also a self‐construction of victimhood. As Vroman et al^51^ put it: “this struggle of being maligned, subjected to pity and having culpability inferred is internalized as shame that results in a devaluing of the self, all of which challenges [their] moral being” (p. 985).

When they took the old implant out they had to remove some bone and muscle because of the poisoning

The type of language used within the texts to describe secondary damage caused as a result of the failure of the ASR has an additional resonance because most of it appears as everyday speech. One person is reported as saying “I then had a second operation and needed a bone graft, several screws and my femur cracked during the op, so needed wiring.” The implications of this secondary damage are made starkly evident to the reader through the words, in short, emphatic sentences, of another damaged ASR‐recipient, “I couldn't walk. I had to live in the lounge. I didn't know if I was ever going to be able to walk again.” The use of emotive language and direct speech helps to create a form of victimhood that is borne bravely and is attributable to an external cause. In one broadsheet publication, the case is firmly made that, in words attributed to two people, the problem was as a result of the ASR. One is quoted as saying “… the tissue damage makes me a classic ASR case. I think the company and the regulator have been negligent” and when another describes the consequences she suffered she is clear about who is to blame:

When they opened me up in March this year, the metal‐on‐metal corrosion (sic) meant most of my pelvis and surrounding tissue had been eaten away; this was nothing to do with the surgery, just the ASR implant

I feel like an 80 year old rather than a 40 year old

Another element in the sequence of problem‐recognition is linked to the emergence of disability and its accompanying losses, physical, social and psychological. The use of first‐person accounts in the texts serves to personalize readers’ understandings of the damaging effects of the failing hip replacement and appeals to them to make empathic links between the experience of those portrayed and their own lives. The accounts include stories of being unable to work, of feeling “robbed of my life,” of life being “put on hold” and of feeling much older than their chronological age. Important life events are disrupted, the wedding of one hip‐recipient is delayed and a young mother talks about not being able to carry her eight week old baby. As another ASR‐recipient puts it, “psychologically it's devastating. Two and a half months after the operation I'm still walking with a stick.”

I’m sure it’s the high levels of cobalt and chromium in my blood but no one can tell me the long‐term affects (sic) of this

The difficulty of facing multiple uncertainties was evident in all the texts. ASR‐recipients worry about the, as yet unknown, long‐term health consequences of exposure to metal ions, and the anxiety provoked by unexplained symptoms. Others fear the possibility of further surgical revision and the effect this could have to later life: “I'm terrified of ending up in a wheelchair. My entire life is on hold. I was offered a top job but had to turn it down because I need revision surgery and I don't know how it will go.”

The loss of sense of self and ability to fulfil desired roles is portrayed within the texts in ways that stress the disruption to lives and construct an ultimate victimhood. The future depicted for damaged ASR‐recipients remains opaque and full of an anxiety that appears to define their lives and render them powerless. These representations of the self, reflect Parsons’ “sick role”^52^ which requires the “sick” person to behave in specific ways, taking and acting on medical advice, seeking to regain health as swiftly as possible and, in exchange, being excused from all usual roles and responsibilities. The construction of victimhood in the accounts seems to depend on the unexceptional nature of hip recipients lives, their previous health, their pain/disability and biomedicine's failure to fulfil its promise of rendering them again a “clean and proper body” (p. 32).^53^ This two‐dimensional construction of hip recipients situates them as relieved of moral responsibility for their suffering although also positioned as in need of recompense.

## 8 CONSTRUCTION OF THE IDENTITIES OF OTHER KEY PLAYERS IN THE ASR STORY

Each damaged ASR‐recipient's story is framed within a network of contextual arrangements relating to other individuals or groups whose positioning play a key role in the story of the ASR.

### 8.1 Blame and accountability

Blame and a demand for accountability are woven throughout all the texts although the ways in which such representations are constructed varies in each publication. This variation is perhaps best explained by political/ideological affiliations, editorial influence or agendas related to existing interests and projects. The Telegraph group, for example, has a history of collaboration with the British Medical Journal in undercover investigations. The Independent reporting adopts a subtle and tangential way of constructing blame, employing the voices of multiple “experts” and coaxing the reader towards a construction of blame of the UK medical device regulator (MHRA). The Daily Mail/MailOnline and the Daily Mirror both strategically mobilize the voice of experts through use of such phrases as “experts said” or “in scientific tests” to present a “factual” account of events surrounding failure of the ASR. Both tabloid publications place special emphasis on the financial aspects of the ASR failure, both to the NHS and to individuals and highlight legal routes to obtaining compensation for suffering.

In all texts, those variously positioned as blameworthy or responsible in accordance with each publication's political agenda, were also accorded a voice. In most cases, this voice was represented through a press release or a spokesperson and quoted verbatim. A self‐justifying tone could often be discerned, for example a spokesperson for the UK medical device regulator (MHRA) who emphasized that, as DePuy had belatedly initiated the recall of the ASR, there was “no need for the MHRA to enforce one.” However, among all the texts there is a noticeable absence. The orthopaedic surgeons who selected and implanted the resurfaced hips and, in some cases were unaware of the device's potential failure when ASR‐recipients began to report pain and discomfort to them, are mentioned only briefly and do not appear to have blame conferred upon them. This absence is paradoxical given medicine's traditional role in managing illness, maintaining quality and patient safety and specifically, in the case of the ASR, orthopaedic surgeons’ role in its selection and implantation. Such discursive strategies adopted in the texts reinforce the press's power to frame the way in which news is presented as well as its power to create polarities, to influence public policy and, ultimately public behaviour.^54^

### 8.2 Media‐given voice

#### 8.2.1 The legal and the medical voice

Within each of the texts, specific voices appear to be intentionally foregrounded. The voice most clearly audible in twelve of the thirteen reports is that of the legal profession. Unusually, the voice accorded to this group is often presented in a manner most often used by the medical profession who in these texts are almost completely silent. Where they are mentioned they are simply “doctors/surgeons” who made decisions for hip recipients. Only one doctor, the Editor of the British Medical Journal, is accorded an “expert” voice. Solicitors on the other hand are made totally visible, their names and the names of their employer are given.

The legal voice in these texts reflects the medical/caring voice in giving both technical and medical advice and information, for example one solicitor is quoted as saying:

We always recommend to our clients the importance of going back to their consultant first for a review of their hip replacement and to ask for an analysis of cobalt and chromium in the blood and serum and, if appropriate, MRI scans even if they are asymptomatic

They express concern and talk in apparently knowledgeable voices about how hip recipients might be affected, psychologically and physically:

It’s had a terrible price on my clients psychologically. It’s broken up relationships and some clients have said they would consider taking their own life because of the pain.

By positioning themselves on the intersection between law and medicine, solicitors’ role becomes ambiguous although they potentially gain an additional measure of public acceptance and trust through the adoption of the medical voice. It is only through connection with the legal profession that hip recipients appear to resist a construction of victimhood. What they require changes from medical intervention to legal rescue and redress through a caring and compassionate legal profession.

## 9 DISCUSSION

This paper explores the role of the UK press media in framing medically induced harm through the reported experiences of damaged hip recipients. In particular, our analysis suggests that the press coverage of the ASR failure was framed through the development of particular representations which located hip recipients (present) experiences within a historically emerging process. It might be argued that any understanding of such experiences is “nonsensical unless it can be linked in some fashion with [the] past” (p. 255).^55^ Suddenly, revealing a sense of hip recipients experiences of severe pain might be “mere whimsy” unless such representations can be “attached to a temporal context revealing their genesis” (ibid.). In this context, what is interesting in exploring here is (a) the ways in which such representations were played out and (b) how these representations also shaped the development of an overarching legal discourse related to the ASR hip recipients accounts of blame and accountability.

Two narratives were particularly important in framing press media coverage of the ASR case: the role of patients as passive recipients of care and a distinction between health and disability identities as related to how individuals’ narratives about the past shaped their sense of present and future. Firstly, our analysis illustrates how individuals were depicted as essentially passive recipients of medical diagnosis and treatment services.^56^ Although patients/the public are the focus of health care, these representations contribute to the traditional conceptualization of patients’ role as passive and non‐contributory participants. These representations provided a key narrative within all the texts and facilitated the development of particular frames of reference that press media used to construct meanings of hip recipients experiences of living with pain. These included narratives of disability for damaged ASR‐recipients, uncertainty and fear about potential future harms (for example as progression to severe pain) and the development of risk perceptions surrounding the failure of the ASR (as inevitable decline).

A strategic way damaged hip recipients stories become active in the production of meanings reflects the ways in which versions of the past become taken for granted and reflected in “ordinary” unexceptional lives. Before the need for hip replacement surgery, representations of “ordinary” lives were evident in the individuals’ narratives in their stories of an active and healthy past. Stories of previous achievements were illustrated to emphasize hip recipients “ordinariness” through the use of personal and family details to which the reader is invited to relate. Following hip replacement surgery, a new narrative was required and thus emerged, particularly one that would polarize hip recipients identities between “healthy/past” and “disability/present” in such a way that were consistent with hip recipients experiences of living with pain. In all texts, hip recipients generally reported that their pain affected most aspects of their lives. Simple tasks had become challenging and they could no longer participate in activities they enjoyed. Moreover, within all the texts, representations of uncertainty and fear related to the, as yet unknown, long‐term consequences of the ASR failure emerged.

However, in all stories what was perceived to be unrelenting pain before the need for surgery remains silent. Although individuals must have been in persistent chronic pain for an extended period of time before their surgery, no details are reported to illustrate how they were living lives constricted by pain. This is quite surprising given that an unmanageable pain is the primary reason for receiving a joint replacement.^57^, ^58^ In so doing, a collective identity has been ascribed to hip recipients as one of activity and vitality, suppressing the very reasons why these patients require hip replacement surgery in the first place. These accounts of hip recipients’ identities as previously healthy and active, constitute them as “genuine” but also further contribute to frame them as victims of iatrogenic harm.

Those whose stories of harm are told within the press texts become also subject to discourses of victimhood and self‐pity. In so doing, they contribute to the construction of blame and responsibility related to other individuals or groups who are key players in the story of the ASR. Each publication follows a different blaming strategy reflecting its political and editorial agenda; however, a core feature of the ASR press coverage is medicine's absence. In all cases, the voice of the orthopaedic surgeons responsible for the selection and implantation of the ASR devices remains silent. This absence is paradoxical given medicine's traditional role in managing illness, maintaining quality and patient safety and specifically, in the case of the ASR, orthopaedic surgeons’ role in its selection and implantation. Moreover, such silent attribution to medicine enables the representation of medicine/orthopaedics as vulnerable, acted upon and in need of protection rather than as having agency to act in their own interests. Instead, litigation comes to the fore as an efficient and reliable way of providing both accountability and retribution for damaged hip recipients/harm arising from medical error. In so doing, the legal voice replaces the medical discourse of “caring” by providing both technical and medical advice and related information to the “victims” of faulty hip replacement surgery to recover damages including medical bills, pain and suffering, and contingent costs such as loss of work capability. By attributing them an active voice the press media portrays legal professionals as having special knowledge and experience dealing with the current flood of lawsuits regarding defective hip replacement devices. At the same time, the enrolment of legal discourse to the stories of the damaged hip recipients prepares them for the potential issuing of proceedings in court to recover compensation over “defective” implants.

Despite, the size and impact of medically induced harm in the UK,^2^ press media coverage of such incidents is significantly less common than coverage of any other patient safety issues and public health debates.^32^ This study aims to contribute to the evidence base on how public discourse on medically induced harm becomes framed through the reported experiences of individuals in press media and also how this process influences the legitimacy of various solutions to medical errors or unanticipated outcomes.

The data presented here are taken for the period between August 2010, when the ASR was withdrawn by the manufacturer, and the end of March 2014 when reports related to the ASR had become less prominent. It is possible that a longer time frame would have allowed for the identification of more diverse representations of medically induced harm for damaged hip recipients. Our analysis included only print news from the UK, and therefore, cannot be taken as representative of the wider media's role in representations of damaged hip recipients. Further research is needed to explore the extent to which media representations of medically induced harm affects levels of trust in health care.

In summary, this study illustrated the ways in which the media portray themselves as espousing the cause of individuals or groups with common iatrogenic experiences and supporting them through the process of attributing blame. Press reports may mobilize public opinion, help set policy agendas and consequently influence political decisions, demonstrating the “general shift in power and social influence from professional groups, including medicine, towards the media” (p. 81).^59^ However, the press's choice of what news to report, always selective and ideological, may in fact reflect different agendas and consequently may be instrumental in mediating the experience of the public^19^ rather than acting on behalf of those harmed. Within the thirteen press texts, the role of law is portrayed as aiding harmed individuals to negotiate recompense for their suffering.

## 10 CONCLUSION

It is well acknowledged that the press media play an integral part in shaping public opinion.^60^ They serve as powerful modes of communication across a large, anonymous, diverse audience where readers and the public can identify with an “imagined community.”^16^, ^61^ In this context, press media may offer a discursive space where messages about trust, fear, risk and blame are conveyed.^62^ Trust in health care is vital because of the vulnerability and uncertainty that illness represents.^63^ It is underpinned by the belief that others will act benignly rather than maliciously and with “beneficence, fairness and integrity” (p. 92).^64^ Health‐care systems are part of the social fabric of society^65^ with which most people must interact at some point in their lives. Changes in societal attitudes in the twenty‐first century, at a time of increased anxiety, risk‐perception and with high demands for accountability, make individual/societal trust in health‐care systems both desirable but also fragile.^66^ Press discourses of blame and accountability of organizations surrounding health care and of the vulnerability and victimhood of hip recipients damage and undermine this trust at a time when for people with health needs trust is essential.

## References

[1] Illich I . Limits to Medicine: Medical Nemesis. London, UK: Penguin; 1976.

[2] Department of Health . An Organisation with a Memory: Report of an Expert Group on Learning from Adverse Events in the NHS (Chaired by the Chief Medical Officer). London, UK: Stationery Office; 2000.

[3] National Joint Registry for England Wales and Northern Ireland . *14th Annual Report* . 2017. https://www.hqip.org.uk/media_manager/public/253/NJR/NJR%2014th%20Annual%20Report%202017.pdf. Accessed February 24, 2018.

[4] Skinner J , Kay P . Commentary: metal on metal hips. Br Med J. 2011;342:d3009. 21572137 10.1136/bmj.d3009

[5] Johnson E , Horwood J , Gooberman‐Hill R . Conceptualising time before surgery: the experience of waiting for hip replacement. Soc Sci Med. 2014;116:126‐133. 24997442 10.1016/j.socscimed.2014.06.037 PMC4124516

[6] Pearce KJ . Media and mass communication theories In: Littlejohn SW, Foss KA, eds. Encyclopedia of Communication Theory. Thousand Oaks; CA: Sage; 2009:623‐627.

[7] Foucault M . Power/Knowledge. Brighton, UK: Harvester; 1980.

[8] Gergen KJ . An Invitation to Social Construction. London, UK: Sage; 1999.

[9] Fairclough N . Media Discourses. London, UK: Bloomsbury Academic; 1995.

[10] Goffman E . Frame Analysis. New York, NY: Free Press; 1974.

[11] Snow DA , Rochford EB Jr , Worden SK , Benford RD . Frame alignment processes, micromobilization, and movement participation. Am Sociol Rev. 1986;51:464‐81.

[12] Entman RM . Framing: toward clarification of a fractured paradigm. J Commun. 1993;43(4):51‐55.

[13] Kitzinger J . Framing and frame analysis In Devereux E, ed. Media Studies: Key Issues and Debates. London, UK: Sage; 2007:134‐161.

[14] Kristiansen CM , Harding CM . Mobilization of health behavior by the press in Britain. Journal Q. 1984;61(2):364‐70, 398.

[15] Nelkin D . Selling Science. How the Press Covers Science and Technology. New York, NY: WH Freeman and Co; 1995.

[16] Seale C . Health and media: an overview. Sociol Health Illn. 2003;25(6):513‐531. 12919443 10.1111/1467-9566.t01-1-00356

[17] Gorini G , Currie L , Spizzichino L , Galeone D , Lopez MJ . Smoke‐free policy development in Italy through the legislative process of the ban 2000–2005, and press media review 1998–2008. Ann Ist Super Sanita. 2011;47:260‐265. 21952150 10.4415/ANN_11_03_04

[18] Hilton S , Hunt K , Langan M , Bedford H , Petticrew M . Newsprint media representations of the introduction of the HPV vaccination programme for cervical cancer prevention in the UK (2005–2008). Soc Sci Med. 2010;70:942‐950. 20064682 10.1016/j.socscimed.2009.11.027 PMC2835855

[19] Bury M , Gabe J . Television and medicine. Medical dominance or trial by media? In Gabe J, Kelleher D, William G, eds. Challenging Medicine *. * London, UK: Routledge; 1994:65‐83.

[20] Dorfman L , Wallack L , Woodruff K . More than a message: framing public health advocacy to change corporate practices. Health Edu Behav. 2005;32:320‐336. 10.1177/1090198105275046 15851542

[21] Wallack L , Dorfman L , Jernigan D , Themba M . Media Advocacy and Public Health: Power for Prevention. Newbury Park, CA: Sage; 1993.

[22] Walsh‐Childers K . Newspaper influence on health policy development. Newspaper Res J. 1994;15:89‐104.

[23] Grilli R , Freemantle N , Minozzi S , Domenighetti G , Finer D . Mass media interventions: effects on health services utilisation. *The Cochrane* . Library. 1999;Issue:4. 10.1002/14651858.CD000389 10796539

[24] Winsten JA . Science and the media: the boundaries of truth. Health Aff. 1985;4(1):5‐23. 10.1377/hlthaff.4.1.5 3997047

[25] Nelkin D . Journalism and science: the creative tension In: Moore M, ed. Health Risks and the Press. Washington, DC: Media Institute; 1989:53‐71.

[26] Hilton S , Patterson C , Teyhan A . Escalating coverage of obesity in UK newspapers: the evolution and framing of the ‘obesity epidemic’ from 1996 to 2010. Obesity. 2012;20:1688‐1695. 22318314 10.1038/oby.2012.27 PMC3408646

[27] Andsager JL , Powers A . Framing women's health with a sense‐making approach: magazine coverage of breast cancer and implants. Health Commun. 2001;13(2):163‐185. 11451103 10.1207/S15327027HC1302_3

[28] Lawrence RG . Framing obesity: the evolution of news discourse on a public health issue. Press/Politics. 2004;9(3):56‐75.

[29] Terkildsen N , Schnell FI , Ling C . Interest groups, the media, and policy debate formation: an analysis of message structure, rhetoric, and source cues. Polit Commun. 1998;15:45‐61.

[30] Vasterman LM , Ruigrok N . Pandemic alarm in the Dutch media: Media coverage of the 2009 influenza A (H1N1) pandemic and the role of the expert sources. European Journal of Communication. 2013;28(4):436‐453.

[31] Nerlich B , Halliday C . Avian flu: the creation of expectations in the interplay between science and the media. Sociol Health Illn. 2007;29(1):46‐65. 17286705 10.1111/j.1467-9566.2007.00517.x

[32] Wallis P , Nerlich B . Disease metaphors in new epidemics: the UK media framing of the 2003 SARS epidemic. Soc Sci Med. 2005;60:2629‐39. 15814187 10.1016/j.socscimed.2004.11.031 PMC7117051

[33] Hinchcliff R , Westbrook J , Greenfield D , Baysari M , Moldovan M , Braithwaite J . Analysis of Australian newspaper coverage of medication errors. Int J Qual Health Care. 2012;24(1):1‐8. 22117025 10.1093/intqhc/mzr067

[34] Anderson J , Neary F , Pickstone J . Surgeons, manufacturers and patients. Basingstoke, UK: Palgrave Macmillan; 2007.

[35] Learmonth I , Young C , Rorabeck C . The operation of the century: total hip replacement. The Lancet. 2007;370:1508‐1519. 10.1016/S0140-6736(07)60457-7 17964352

[36] Daniel J , Pynsent P , McMinn D . Metal‐on‐metal resurfacing of the hip in patients under the age of 55 years with osteoarthritis. J Bone Joint Surg. 2004;86(2):177‐184. 10.1302/0301-620x.86b2.14600 15046429

[37] Cohen D . Out of joint: the story of the ASR. Br Med J. 2011;342:d2905. 21572134 10.1136/bmj.d2905

[38] Wienroth M , McCormack P , Joyce T . Precaution, governance and the failure of medical implants: the ASR ^TM^ hip in the UK. Life Sci Soc Policy. 2014;10(1):19. 26573983 10.1186/s40504-014-0019-2 PMC4480348

[39] Langton DJ , Sidaginamale RP , Joyce TJ , Natu S , Blain P , Jefferson RD , Rushton S , Nargol AVF . The clinical implications of elevated blood metal ion concentrations in asymptomatic patients with MoM hip resurfacings: a cohort study. BMJ Open. 2013;3:e001541. 10.1136/bmjopen-2012-001541 PMC3612810 23482990

[40] Mao X , Wong A , Crawford R . Cobalt toxicity – an emerging clinical problem in patients with metal‐on‐metal hip prostheses? Med J Aust. 2011;194:649‐651. 21692725 10.5694/j.1326-5377.2011.tb03151.x

[41] Cohen D . How safe are metal‐on‐metal hip implants? Br Med J. 2012;344:e1410. 22374741 10.1136/bmj.e1410

[42] National Joint Registry for England Wales and Northern Ireland . *15th Annual Report* . 2018 http://www.njrcentre.org.uk/njrcentre/Reports-Publications-and-Minutes/Annual-reports. Accessed October 22, 2018.

[43] National Institute for Health and Care Excellence . Total hip replacement and resurfacing arthroplasty for end‐stage arthritis of the hip. 2014 https://www.nice.org.uk/guidance/ta304/resources/total-hip-replacement-and-resurfacing-arthroplasty-for-endstage-arthritis-of-the-hip-pdf-82602365977285. Accessed October 22, 2018.

[44] Neuendorf K . The Content Analysis Guidebook. Thousand Oaks, CA: Sage Publications; 2002.

[45] Fox N . Postmodernism, Sociology and Health. Buckingham, UK: Open University Press; 1993.

[46] Jeffrey R . Normal rubbish: deviant patients in casualty departments. Sociol Health Illn. 1979;1(1):90‐107. 10250394 10.1111/1467-9566.ep11006793

[47] Martin D . Too polite to make a fuss: Elderly NHS patients suffer in silence say health watchdog. *Mail Online* 2014 http://www.dailymail.co.uk/news/article-2598355/Too-polite-make-fuss-Elderly-NHS-patients-suffer-silence-say-health-watchdog.html. Accessed February 24, 2018.

[48] Bioethics JN . Embodied ethics: from the body as specimen and spectacle to the body as patient In: Mascia‐Lees F, ed. A Companion to the Anthropology of the Body and Embodiment. Chichester, UK: Blackwell Publishing; 2011:72‐85.

[49] Williams S , Bendelow G . Emotions, health and illness: the ‘missing link’ in medical sociology? In: James V, Gabe J, eds. Health and the Sociology of Emotions. Oxford, UK: Blackwell; 1996:25‐53.

[50] Dow C , Roche P , Ziebland S . Talk of frustration in the narratives of people with chronic pain. Chronic Illn. 2012;8(3):176‐191. 22473060 10.1177/1742395312443692

[51] Vroman K , Warner R , Chamberlain K . Now let me tell you in my own words: narratives of acute and chronic low back pain. Disabil Rehabil. 2009;31(12):976‐987. 19037775 10.1080/09638280802378017

[52] Turner B . Medical Power and Social Knowledge. London, UK: Sage; 1995.

[53] Shildrick M . Corporeal cuts: surgery and the psycho‐social. Body Soc. 2008;14(1):31‐46.

[54] Nelkin D . An uneasy relationship: the tensions between medicine and the media. Lancet. 1996;347:1600‐03. 8667872 10.1016/s0140-6736(96)91081-8

[55] Gergen KJ , Gergen MM . Narratives of the self In: Sarabine TR, Scheibe KE, eds. Studies in Social Identity. New York, NY: Praeger; 1983:254‐273.

[56] Armstrong D . Actors, patients and agency: a recent history. Sociol Health Illn. 2014;36(2):163‐174. 24372176 10.1111/1467-9566.12100

[57] Norman‐Taylor FH , Palmer CR , Villar RN . Quality‐of‐life improvement compared after hip and knee replacement. J Bone Joint Surg Br. 1996;78(1):74‐77. 8898131

[58] Robertsson O , Dunbar M , Pehrsson T , Knutson K , Lidgren L . Patient satisfaction after knee arthroplasty: a report on 27,372 knees operated on between 1981 and 1995 in Sweden. Acta Orthop. 2000;71(3):262‐267. 10.1080/000164700317411852 10919297

[59] Gabe J , Bury M . Halcion nights: a sociological account of a medical controversy. Sociology. 1996;30(3):447‐469.

[60] Gamson WA , Modigliani A . Media discourse and public opinion on nuclear power: a constructionist approach. Am J Sociol. 1989;95(1):1‐37.

[61] Anderson B . Imagined Communities: Reflections on the Origin and Spread of Nationalism, 2nd edn London, UK: Verso; 1991.

[62] Oaks L . The politics of health risk warnings: social movements and controversy over the link between abortion and breast cancer In: Harthorn BH, Oaks L, eds. Risk, Culture and Heath Inequality: Shifting Perception of Danger and Blame. Westport, CT: Greenwood; 2003:79‐101.

[63] Calnan M , Rowe R . Trust matters in health care. Maidenhead, UK: Open University Press; 2008.

[64] Calnan M , Sanford E . Public trust in health care: the system or the doctor? Qual Saf Health Care. 2004;13:92‐97. 15069214 10.1136/qshc.2003.009001 PMC1743818

[65] Gilson L . Trust and the development of health care as a social institution. Soc Sci Med. 2003;56:1453‐1468. 12614697 10.1016/s0277-9536(02)00142-9

[66] Elston M . Remaking a trustworthy medical profession in twenty‐first‐century Britain? In: Gabe J, Calnan M, eds. The New Sociology of the Health Service. Hoboken, UK: Taylor and Francis; 2009:17‐36.

